# Ireland: Europe's outlier in primary health care

**DOI:** 10.1016/j.lanepe.2025.101253

**Published:** 2025-03-03

**Authors:** The Lancet Regional Health – Europe

Despite being one of the richest countries in Europe, Ireland is the only European country without universal coverage of primary health care. Although substantial investments have been made in health through the government budget—with public health spending increasing from €15.3 billion in 2018 to €22.8 billion in 2024—58% of residents do not have access to free primary health care. Ireland's primary health-care model stands in stark contrast to other European countries, where the costs of general practitioner (GP) visits are either fully or partially covered, for the whole population, through public health spending or compulsory health insurance.

The heart of Ireland's primary health care dilemma lies in its two-tier system: Category 1 comprises individuals who qualify for free primary care through medical cards or GP visit cards, typically based on their age, very low-income (e.g., individuals aged <65 years without dependents earning <€184 a week), or chronic health conditions; Category 2 includes individuals who do not qualify for these cards, encompassing many low-income and middle-income earners for whom primary care costs can be a significant burden. Larger households might appear to have a high total income but face substantial expenses for essential care. As of November, 2024, Category 1 covered only about 42% of the population. For everyone else, the cost of a GP visit can remain prohibitively high, ranging from €50 to 80 per appointment in urban areas—substantially higher than costs associated with primary care visits in other European countries—with no publicly financed coverage available.

The situation has substantial consequences. Publicly financed inpatient care and the medical card scheme help prevent high levels of catastrophic health spending in Ireland, with only around 1.2% of households experiencing such costs in 2016—a figure lower than the European average. However, this statistic does not fully capture access barriers, as about 25% of the population delay GP visits due to cost concerns. Early diagnosis and intervention are crucial for reducing the long-term burden of disease, which can only be achieved when individuals can afford to seek medical attention at the first sign of illness.

Successive governments have recognised the damage caused by inadequate access to primary care and proposed reforms such as the Sláintecare initiative, which aims to establish universal access to health care based on medical need rather than financial means. A 2018 national survey in Ireland showed that 87% of respondents supported the implementation of universal health care. Expanding free GP care to the entire population is a necessary investment, estimated to range from €381 million to €881 million if implemented in 2026—a fraction of the amount allocated annually for health spending. However, financial constraints are not the only obstacle; there is also a looming shortage of GPs, with a fifth of the current workforce aged older than 60 years and many expected to retire in the next decade.

The inaccessible primary care system also places tremendous strain on hospitals. Despite the Irish Government's record health-care spending for 2025, in January 2025, 13,972 individuals had to be treated without a hospital bed due to overcrowding. Improving primary care accessibility could help alleviate this strain by providing treatment at an earlier stage, preventing them from escalating into emergencies.

Health-care spending is ultimately a political choice, and the Irish Government must prioritise this issue. The current system, which places the financial burden on individuals rather than the state, is misaligned with Ireland's economic standing and European norms. It is time that Ireland moves from being an outlier in Europe to providing universal access to primary health care and upholds the commitments it made through the Sustainable Development Goals (2015), particularly goal 3.8, and the European Pillar of Social Rights (2017)—to provide universal health care to all citizens. This transition is not just a moral imperative; it is essential for improving health outcomes, ensuring affordable access to care, optimizing healthcare resources, and safeguarding Ireland's healthcare system for the future.

The real question is not whether the country can afford it, but whether its leaders have the political will to make it happen. It is time for decisive action to create a health-care system that serves all citizens equally.

